# Scanning X-ray nanodiffraction from ferroelectric domains in strained K_0.75_Na_0.25_NbO_3_ epitaxial films grown on (110) TbScO_3_ 1

**DOI:** 10.1107/S1600576717000905

**Published:** 2017-02-17

**Authors:** Martin Schmidbauer, Michael Hanke, Albert Kwasniewski, Dorothee Braun, Leonard von Helden, Christoph Feldt, Steven John Leake, Jutta Schwarzkopf

**Affiliations:** a Leibniz-Institut für Kristallzüchtung, Max-Born-Strasse 2, Berlin 12489, Germany; b Paul-Drude-Institut für Festkörperelektronik, Leibniz-Institut im Forschungsverbund Berlin, Hausvogteiplatz 5–7, Berlin 10117, Germany; c ESRF – The European Synchrotron, 71 Avenue des Martyrs, Grenoble Cedex 9, CS-40220, 38043, France

**Keywords:** X-ray nanodiffraction, ferroelectric domains, K_*x*_Na_1−*x*_NbO_3_, strained epitaxial films

## Abstract

Scanning X-ray nanodiffraction on a highly periodic ferroelectric domain pattern of a strained K_0.75_Na_0.25_NbO_3_ epitaxial layer has been performed by using a focused X-ray beam of about 100 nm probe size. A 90°-rotated domain variant which is aligned along [12]_TSO_ has been found in addition to the predominant domain variant where the domains are aligned along the [12]_TSO_ direction of the underlying (110) TbScO_3_ (TSO) orthorhombic substrate. Owing to the larger elastic strain energy density, the 90°-rotated domains appear with significantly reduced probability. Furthermore, the 90°-rotated variant shows a larger vertical lattice spacing than the 0°-rotated domain variant. Calculations based on linear elasticity theory substantiate that this difference is caused by the elastic anisotropy of the K_0.75_Na_0.25_NbO_3_ epitaxial layer.

## Abstract 2

Scanning X-ray nanodiffraction with 100 nm spatial resolution has been applied to investigate the ferroelectric domain structure of K_0.75_Na_0.25_NbO_3_ epitaxial layers grown on a (110) TbScO_3_ substrate using metal–organic chemical vapour deposition. Two variants differing in domain wall alignment and vertical lattice parameters have been identified and independently analysed.

## 1. Introduction   

Epitaxial growth of thin films enables control of ferroelectric properties or even provides the evolution of ferroelectric phases which do not exist in the corresponding bulk phases. For example, theoretical (Sheng *et al.*, 2008 ▸; Bai & Ma, 2010 ▸; Koukhar *et al.*, 2001 ▸) and experimental (Chen *et al.*, 2011 ▸; Zeches *et al.*, 2009 ▸; Everhardt *et al.*, 2016 ▸) work has revealed that formation of ferroelectric monoclinic phases can be induced *via* application of epitaxial strain, in particular when the strain is anisotropic (Sheng *et al.*, 2008 ▸; Bai & Ma, 2010 ▸; Koukhar *et al.*, 2001 ▸). Ferroelectric monoclinic phases are of fundamental interest owing to the ability of the electric polarization vector to rotate continuously in a particular symmetry plane of the monoclinic unit cell, potentially leading to giant piezoelectric responses (Fu & Cohen, 2000 ▸; Vanderbilt & Cohen, 2001 ▸; Davis *et al.*, 2006 ▸, 2007 ▸).

The anisotropy in epitaxial strain can also be used to control the crystallographic orientation of the epilayers, which in turn determines the direction of the ferroelectric polarization vector. Strain engineering on various substrates with varying lattice mismatches thus enables tuning of the ferroelectric properties (Schwarzkopf *et al.*, 2012 ▸). In this respect, the perovskite-like compound K_*x*_Na_1−*x*_NbO_3_ is particularly suitable owing to its high piezoelectric coefficients (Saito *et al.*, 2004 ▸; Wang *et al.*, 2014 ▸), anisotropic elasticity coefficients (Kalinichev *et al.*, 1993 ▸) and orthorhombic crystal symmetry.

Anisotropic strain can be induced in K_*x*_Na_1−*x*_NbO_3_ epitaxial layers if they are grown on orthorhombic substrates. In a previous study, performed on K_0.75_Na_0.25_NbO_3_ grown on (110) TbScO_3_, we proved the emergence of an inclined monoclinic *M*_*A*_ phase (Schwarzkopf *et al.*, 2016 ▸) which is associated with both a strong vertical and a strong horizontal electrical polarization component (Vanderbilt & Cohen, 2001 ▸). Along with the observation of the *M_A_* phase, a one-dimensional highly periodic ferroelectric domain pattern is formed which extends over several tens of micrometres. In adjacent domains the in-plane component of the polarization vector periodically changes by 180°. However, besides this prominent domain pattern which is aligned along the lateral 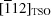 direction of the underlying TbScO_3_ (TSO) substrate (which we refer to as the ‘0° variant’), a structural variant of a 90°-rotated domain pattern is also observed. Here, the unit cells of the epitaxial layer are rotated in-plane by 90°, leading to a corresponding rotation of the one-dimensional domain pattern, which is now aligned along the lateral 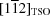 direction. For this 90°-rotated configuration (‘90° variant’) the elastic strain energy density is enhanced compared with the 0°-rotated configuration, leading to a strongly reduced probability (<10%) of appearance.

But, simultaneously, calculations based on linear elasticity also predict a larger vertical strain in the 90° domain variant compared with the 0° domain variant. Since ferroelectric properties like the Curie temperature (*T*_C_) are strongly correlated with the incorporated lattice strain, the emergence of either one or two phases may drastically change the film’s ferroelectric properties on the nanoscale (*e.g.* the temperature or broadness of a phase transition). In this regard, it is highly desirable to verify experimentally the different strain states of the two domain variants for a fundamental understanding of their utility for ferroic applications.

To identify the domain pattern in K_*x*_Na_1−*x*_NbO_3_ epitaxial layers, piezoresponse force microscopy (PFM) and high-resolution X-ray diffraction have frequently been employed (Schwarzkopf *et al.*, 2016 ▸). PFM serves as a local probe technique and images the spatial distribution of the ferroelectric polarization vector with a lateral resolution of typically 10 nm. On the other hand, high-resolution X-ray diffraction is a powerful tool to investigate strain, surface orientation and monoclinic distortion on the submicroscopic or even nanoscopic length scale (Schmidbauer, 2004 ▸; Pietsch *et al.*, 2004 ▸). However, in a conventional X-ray scattering experiment the footprint of the illuminated X-ray beam on the sample is in the region of a few millimetres. This leads to incoherent averaging over structures which show up on a much smaller length scale. In our case, the 90° domain variant appears in areas of typically 1 µm^2^ in size (Schwarzkopf *et al.*, 2016 ▸).

In this paper, we report an advanced X-ray diffraction experiment using a nanofocused monochromatic X-ray beam where high strain sensitivity is combined with spatial resolution in the 100 nm regime. We will show that, for K_0.75_Na_0.25_NbO_3_ epitaxial layers grown on (110) TbScO_3_, both domain variants can be independently identified and analysed. Thereby, the theoretical prediction of a different vertical lattice spacing between the two could be verified and evaluated. The observations are discussed within the framework of calculations based on linear elastic theory.

## 2. Experimental   

K_0.75_Na_0.25_NbO_3_ thin films were grown on (110) TbScO_3_ substrates using liquid-delivery spin metal–organic chemical vapour deposition (MOCVD). Since it is performed close to thermodynamic equilibrium and at high oxygen partial pressures, MOCVD potentially provides highly perfect films with nearly stoichiometric composition and smooth surfaces/interfaces. A detailed description of the growth parameters can be found in the work by Schwarzkopf *et al.* (2012 ▸, 2016 ▸).

The surface morphology of the films was analysed *via* atomic force microscopy (Asylum Research MFP3D stand-alone). The ferroelectric domain state and local piezoelectric responses of the K_0.75_Na_0.25_NbO_3_ films were studied by the PFM setup of the atomic force microscope, utilizing the dual alternating-current resonance tracking (DART) mode (Rodriguez *et al.*, 2007 ▸).

The nanofocus X-ray diffraction experiment was performed on the ID01 experimental station of the European Synchrotron Radiation Facility (ESRF). The principal experimental setup has already been reported by Chahine *et al.* (2014 ▸, 2015 ▸). However, slightly different parameters were used, better adapted to our purposes, leading to the necessity to describe our experimental setup briefly. A 300 µm diameter gold Fresnel zone plate (FZP) with an outermost zone width of 70 nm was employed. At 8 keV X-ray energy (λ = 1.5498 Å) this yields a focal length of about 135 mm for the first diffraction order, which was selected by a 50 µm order-sorting aperture about 20 mm upstream of the sample. The resulting angular divergences are about 2.2 (horizontal, h) × 2.2 mrad (vertical, v). At the same time, the X-ray beam was focused down to a spot size of about 150 (h) × 90 nm (v) at the sample position with an intensity of about 2 × 10^9^ photons s^−1^. The sample was mounted on an *x*-*y*-*z* scanning piezoelectric stage with a resolution of 2 nm and range of 100 µm in all three directions. An optical microscope enabled accurate sample positioning at the centre of rotation of the goniometer.

A fast-readout two-dimensional detector (MAXIPIX; Ponchut *et al.*, 2011 ▸), consisting of 516 × 516 pixels with 55 µm pixel size, was placed at a distance of 986 mm from the sample. This corresponds to a detector angular resolution of about 0.0032° per pixel. Placing the detector closer to the sample (which would adapt the detector angular resolution to the primary beam divergence, which is a factor of 40 larger) would not lead to additional information since the scattered intensity on the detector drops rather quickly in the vicinity of the observed Bragg reflections. Full frame rates of up to 100 Hz can be achieved. The combined use of the FZP together with a fast two-dimensional detector principally allows for (i) three-dimensional reciprocal-space mapping at a selected fixed position in real space, with a resolution in reciprocal space which is limited by the angular resolution of the detector and the divergence of the incident beam, and (ii) scanning X-ray nanodiffraction at a selected fixed position in reciprocal space, with a spatial resolution which is comparable to the focal size of the primary X-ray beam. Additionally, both techniques can be performed in a combined way, leading to five-dimensional data sets. In the present work we focus on scanning X-ray nanodiffraction of the ferroelectric domain patterns.

## 3. Anisotropic strain   

For perovskite-like materials, it is often advantageous to use the pseudocubic (pc) notation, which can be easily obtained from the orthorhombic notation by a simple coordinate transformation. Similar to the notation of Vailionis *et al.* (2011 ▸), the orthorhombic (*a*_o_, *b*_o_, *c*_o_) and pseudocubic (*a*_pc_, *b*_pc_, *c*_pc_, α_pc_) latttice parameters are interconnected *via a*_pc_ = *c*_o_, *b*_pc_ = *c*_pc_ = 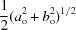 and α_pc_ = 2tan^−1^(*b*_o_/*c*_o_), where α_pc_ is the angle between the *b*_pc_ and *c*_pc_ axes. Throughout the following we will use the pseudocubic notation for the K_*x*_Na_1−*x*_NbO_3_ epitaxial layers, whereas the orthorhombic notation will be used for the TbScO_3_ substrate (*a* = 5.7233, *b* = 5.4543, *c* = 7.9147 Å) (Veličkov *et al.*, 2008 ▸).

**Figure 1:**
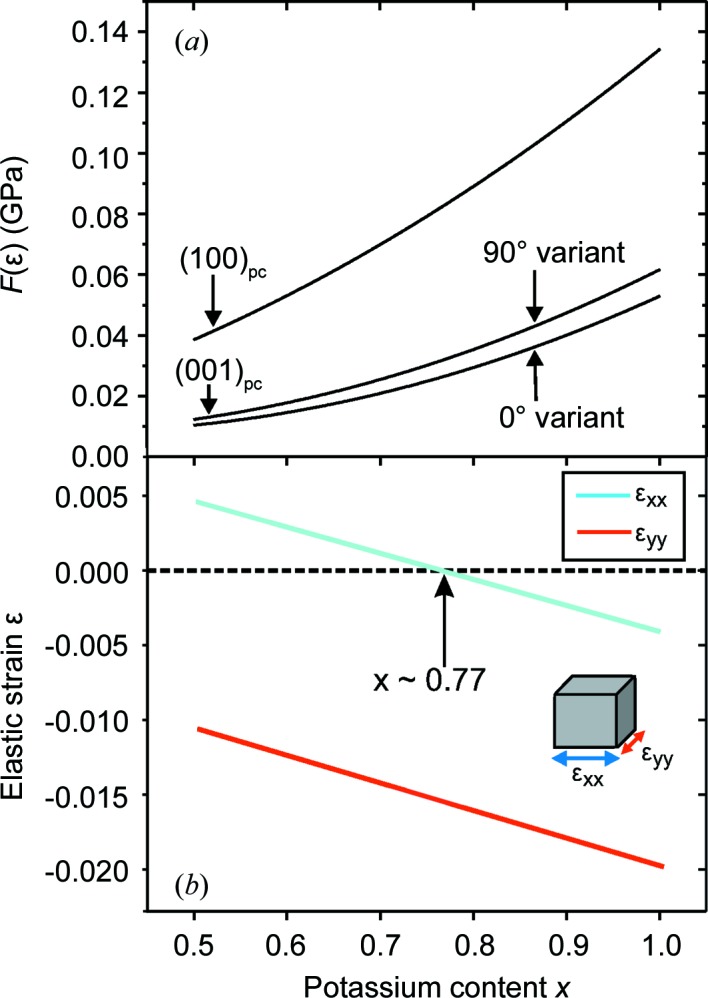
(*a*) Calculated elastic strain energy density *F*(∊) of (100)_pc_ and (001)_pc_ oriented K_*x*_Na_1−*x*_NbO_3_ epitaxial layers grown on (110) TbScO_3_ substrates as a function of potassium content *x*. The two curves for (001)_pc_ differ in their in-plane orientation of the pseudocubic unit cell; for details see text. (*b*) Anisotropic in-plane strains ∊_*xx*_ and ∊_*yy*_ of an (001)_pc_ K_*x*_Na_1−*x*_NbO_3_ thin film grown on a (110) TbScO_3_ substrate as a function of potassium content *x*. Here, the epitaxial relationship between the K_*x*_Na_1−*x*_NbO_3_ film and the TbScO_3_ substrate is assumed to be [100]_pc_ || [001]_TSO_ and [010]_pc_ || 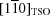.

In Fig. 1 ▸(*a*) the elastic strain energy density *F*(∊) of a K_*x*_Na_1−*x*_NbO_3_ epitaxial layer is displayed as a function of potassium content (*x* > 0.5) for different surface orientations of the pseudocubic unit cell. These calculations are based on the elastic coefficients of KNbO_3_ (Kalinichev *et al.*, 1993 ▸) and they reveal that *F*(∊) is significantly higher for the (100)_pc_ surface orientation than for the (001)_pc_ surface orientation. This energy difference forces the K_*x*_Na_1−*x*_NbO_3_ epitaxial layers to grow on the TbScO_3_ substrate with the (001)_pc_ surface orientation. In this case a strongly anisotropic epitaxial strain emerges, illustrated in Fig. 1 ▸(*b*). For a potassium content of *x* = 0.75, which is applied in this study, the epitaxial strain is highly compressive in one in-plane direction (∊_*yy*_) and very weakly tensile in the corresponding orthogonal direction (∊_*xx*_).

The elastic strain energy density of the (001)_pc_ surface orientation depends on the azimuthal epitaxial relationship of the pseudocubic unit cell on the TbScO_3_ substrate. For the 0° domain variant this is given by [100]_pc_ || [001]_TSO_ and [010]_pc_ || 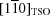, while for the 90° variant it is represented by [010]_pc_ || [001]_TSO_ and [100]_pc_ || 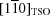. As demonstrated in Fig. 1 ▸(*a*), the elastic strain energy density *F*(∊) is slightly smaller for the 0° variant. The observed energy difference can be attributed to the elastic anisotropy of the epitaxial layer, which is also reflected in a different vertical lattice parameter for each domain variant. The calculations predict a slightly larger vertical lattice parameter for the 90° variant by about (Δ*d*/*d*)_*z*_ = 2.4 × 10^−4^.

## 4. Results and discussion   

**Figure 2:**
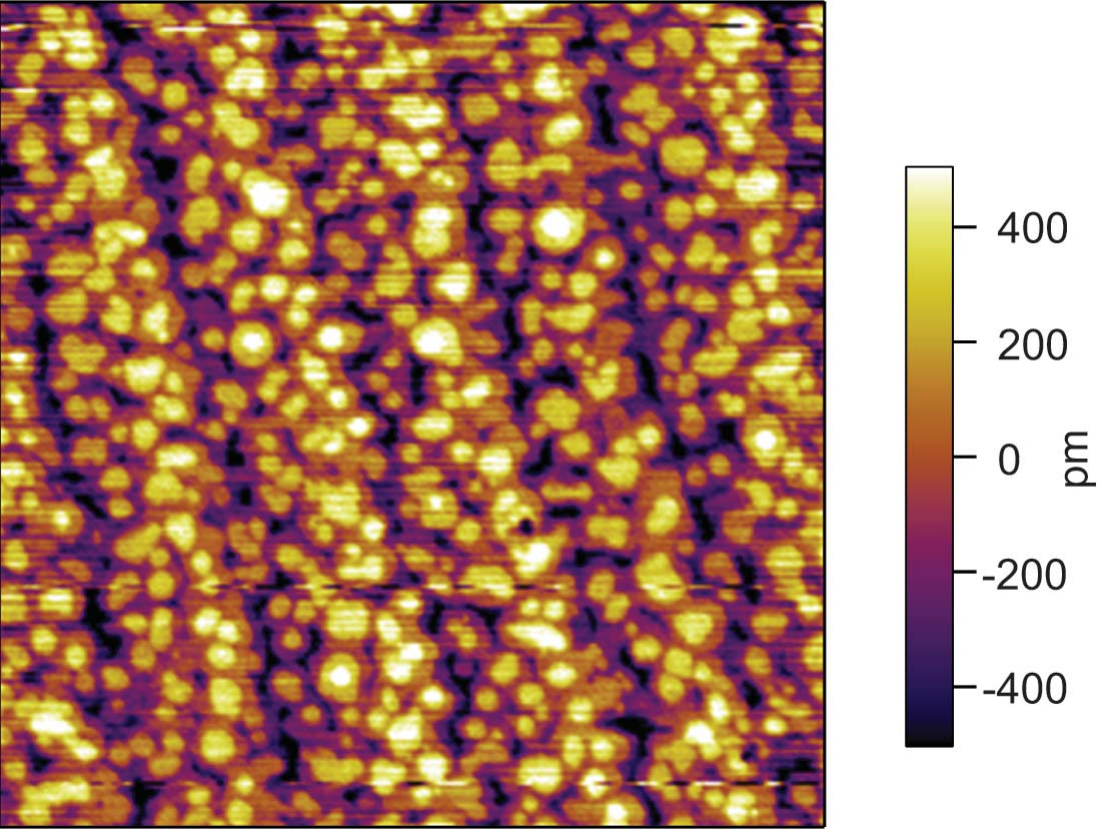
Atomic force micrograph (2 × 2 µm) of a 23 nm K_0.75_Na_0.25_NbO_3_ epitaxial layer grown on a (110) TbScO_3_ substrate.

**Figure 3:**
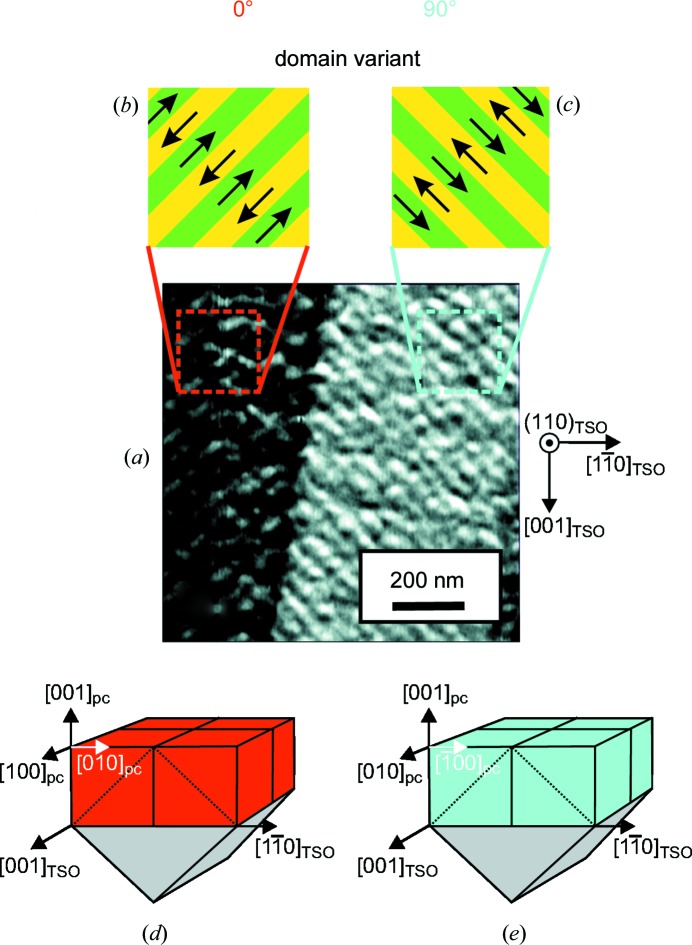
(*a*) Lateral piezoresponse image (1 × 1 µm) of a 23 nm K_0.75_Na_0.25_NbO_3_ epitaxial layer grown on a (110) TbScO_3_ substrate showing a periodic domain pattern with ∼46 nm periodicity. (*b*) The 0° domain variant and (*c*) the 90° domain variant are shown schematically. The corresponding in-plane components of the ferroelectric polarization vector (black arrows) in the individual domains indicate 180° domain walls within one variant. (*d*), (*e*) The epitaxial relationships between the K_0.75_Na_0.25_NbO_3_ epitaxial layer (represented by the pseudocubic unit cells) and the underlying (110) TbScO_3_ substrate for (*d*) the 0° domain variant and (*e*) the 90° domain variant.

The surface morphology of the 23 nm K_0.75_Na_0.25_NbO_3_ epitaxial layer shows atomically smooth terraces about 400 nm broad, which correspond to the 0.05° off-orientation of the TbScO_3_ substrates (Fig. 2 ▸). The root-mean-square surface roughness can be estimated to be about 2 Å. The lateral PFM signal exhibits a well ordered periodic domain pattern (Fig. 3 ▸ *a*). It contains both the 0° (Fig. 3 ▸ *b*) and 90° (Fig. 3 ▸ *c*) variants, which differ in their azimuthal alignment (Figs. 3 ▸ *d* and 3 ▸ *e*). In both cases the domain periodicity can be determined to be about 46 nm. X-ray rocking curves and reciprocal-space maps obtained on a conventional X-ray diffractometer prove that the K_0.75_Na_0.25_NbO_3_ epitaxial layer is coherently grown onto the TbScO_3_ substrate with (001)_pc_ orientation.

**Figure 4:**
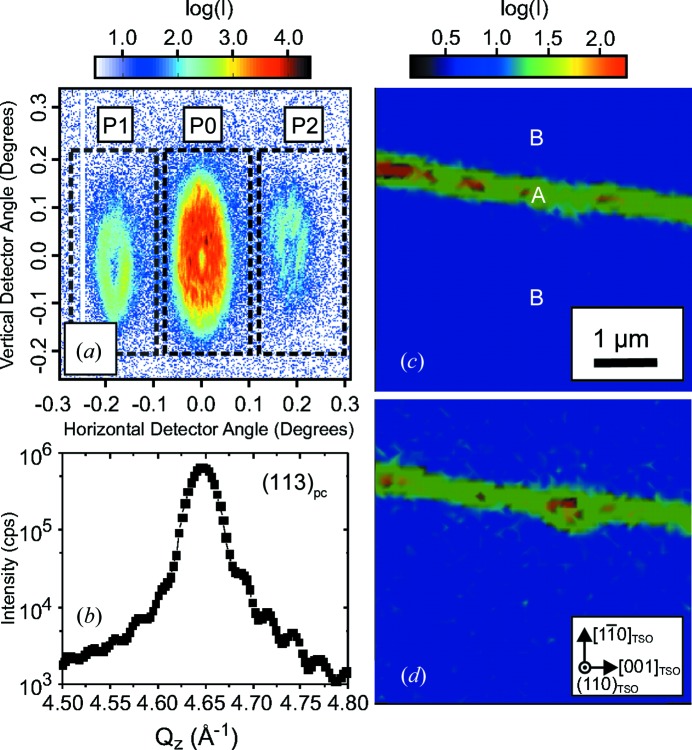
(*a*) A two-dimensional detector frame recorded close to the (113)_pc_ reciprocal lattice vector accumulated at a selected position on the sample. The feature P0 corresponds to the crystal truncation rod of the sample, while P1 and P2 are caused by the lateral periodicity of the domains along 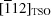. The dashed areas mark the regions of intensity integration. (*b*) The integrated intensity of P0 as a function of the vertical scattering vector component *Q_z_*. (*c*), (*d*) Integrated (113)_pc_ intensity maps (5 × 5 µm) of (*c*) P1 and (*d*) P2 in real space.

Close to the asymmetric coplanar (113)_pc_ reciprocal-lattice point (steep incidence, glancing exit) of the strained K_0.75_Na_0.25_NbO_3_ layer [which appears in the vicinity of the (422)_TSO_ reflection peak of the substrate], a detector frame has been accumulated for a selected position of the X-ray beam on the sample (Fig. 4 ▸ *a*). Near to the strong central scattering feature P0, which we assign to the crystal truncation rod (CTR), two weak side maxima P1 and P2 can be observed, which are caused by the domain periodicity. In the chosen scattering geometry the 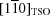 direction is perpendicular to the scattering vector. The scattering geometry is thus sensitive to the 90° domain variant, and the emergence of side maxima shows that the X-ray beam is actually probing this type of domain.

The angular distance, Δ, of P1 and P2 from the CTR (P0) on the detector can be used to determine the domain period *L* by applying the expression *L* = λ/Δ. From Fig. 4 ▸(*a*) a value of Δ = 0.19 ± 0.01° is derived, from which a domain periodicity of *L* ≃ 47 ± 2 nm can be evaluated, which is in very good agreement with the PFM data shown in Fig. 3 ▸(*a*). However, owing to the large convergence of the primary beam all scattering features are remarkably broadened. Nevertheless, they do not overlap and can be analysed independently. The integrated intensity of the CTR [accumulated within the central dashed area of Fig. 4 ▸(*a*)] is displayed in Fig. 4 ▸(*b*) as a function of the vertical scattering vector component *Q_z_*. The intensity distribution shows a broad maximum at *Q_z_* = 4.65 Å^−1^ accompanied by periodic thickness fringes, which prove the coherent growth of the epitaxial layer and smooth interfaces. From the distance between adjacent fringes, a film thickness of *t* = 23 ± 1 nm can be derived, which is in agreement with conventional high-resolution X-ray diffraction curves measured using a large X-ray beam (not shown here).

**Figure 5:**
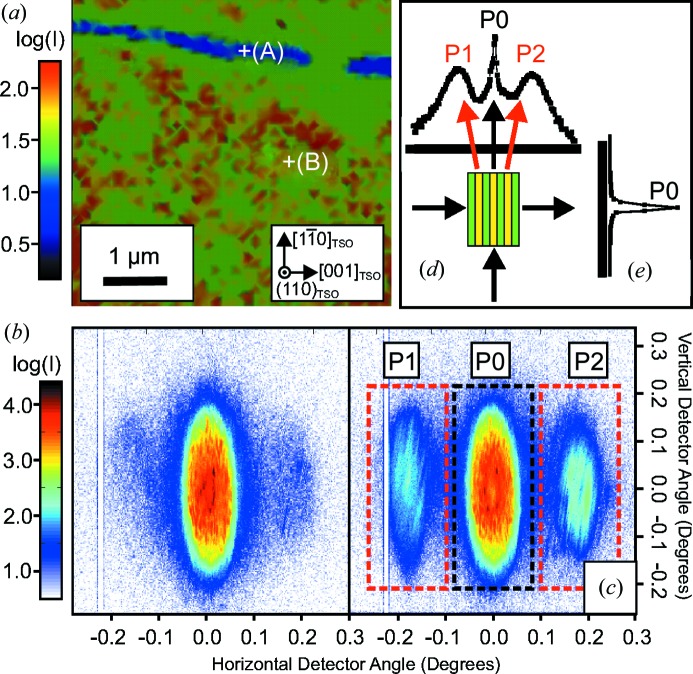
(*a*) Integrated 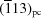 intensity map (5 × 5 µm) of the satellite peak P1. (*b*), (*c*) The corresponding detector frames accumulated at the marked positions in areas A and B, respectively. The dashed areas in panel (*c*) mark regions of intensity integration. (*d*), (*e*) The expected diffraction profiles when the incoming X-ray beam is (*d*) parallel and (*e*) perpendicular to the domain walls.

For *Q_z_* = 4.65 Å^−1^, the integrated intensities of the features P1 and P2 [accumulated within the outer two dashed areas of Fig. 4 ▸(*a*)] were analysed as a function of the position of the X-ray spot on the sample within a 5 × 5 µm area (Figs. 4 ▸ *c* and 4 ▸ *d*, respectively). Two different regions, denoted A and B, can be distinguished. Inside the needle-shaped region A, the intensities of P1 and P2 are strong. By contrast, in the surrounding region B the integrated intensities of P1 and P2 vanish. A complementary behaviour is observed in the vicinity of the asymmetric 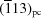 reciprocal-lattice point, for which the sample was rotated by 90° around the surface normal. In this scattering geometry the 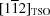 direction is perpendicular to the scattering vector. Similar to the (113)_pc_ Bragg reflection, we observe the scattering features P0, P1 and P2, but the intensities of P1 and P2 are now strong in the surrounding matrix B (Figs. 5 ▸ *a* and 5 ▸ *c*), while the intensities are strongly suppressed within the needle-shaped area A (Figs. 5 ▸ *a* and 5 ▸ *b*).

This observed behaviour can be easily explained. If the plane of incidence of the primary X-ray beam is parallel to the domain walls, satellite peaks caused by the domain periodicity show up in the detector [see schematic presentation in Fig. 5 ▸(*d*)]. On the other hand, if the domain walls are perpendicular to the plane of incidence no satellite peaks can be observed (Fig. 5 ▸ *e*). From our experimental findings we can therefore conclude that the needle-shaped region A can be assigned to the 90° domain variant which appears with reduced probability, while the surrounding matrix can be assigned to the 0° domain variant. However, it must be noted that we know the centre of sample rotation with respect to the X-ray spot with an accuracy of 10 µm for all rotation circles. Without a clear two-dimensional feature in the sample observable in both reflections, we are unlikely to be able to overlap the images perfectly (*cf.* Figs. 4 ▸ and 5 ▸).

**Figure 6:**
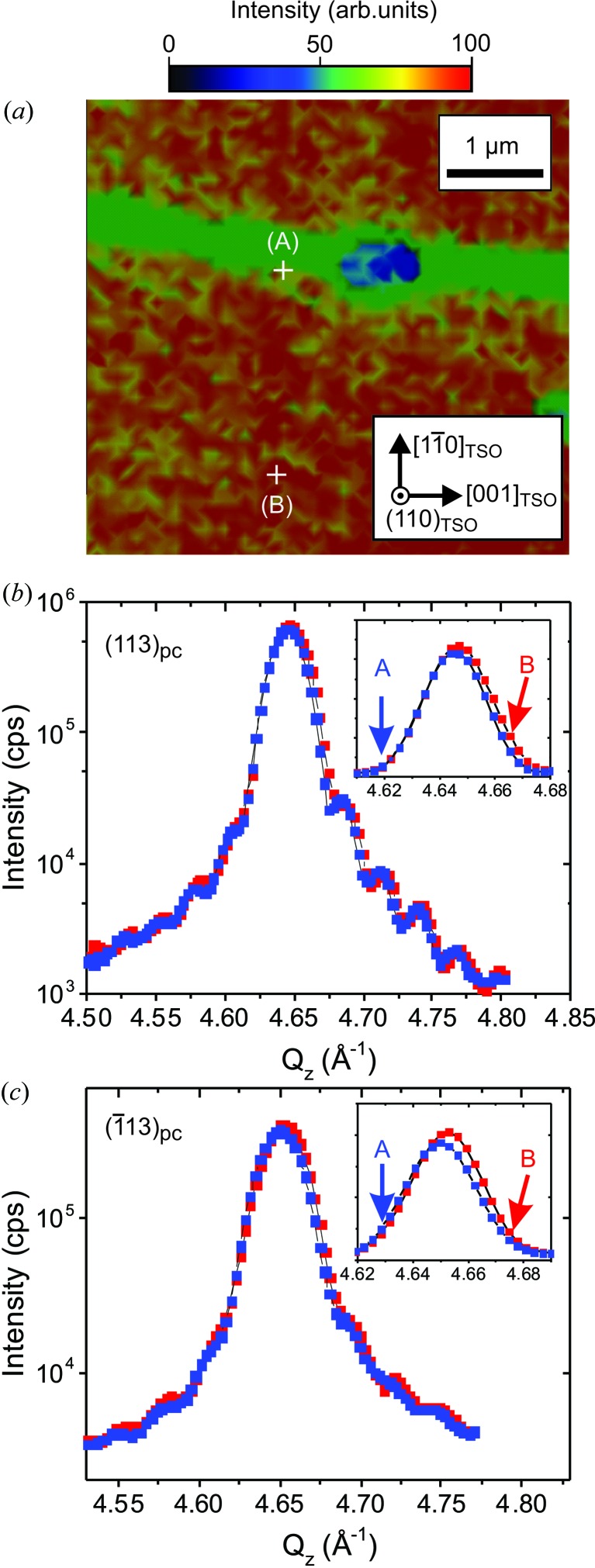
(*a*) Integrated intensity map (5 × 5 µm) of the (113)_pc_ CTR measured at *Q_z_* = 4.65 Å^−1^. (*b*) The corresponding integrated intensity as a function of *Q_z_* measured at the sample positions A (blue symbols) and B (red symbols). (*c*) The corresponding integrated intensity as a function of *Q_z_* in the vicinity of 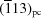. The insets in parts (*b*) and (*c*) are enlargements of the Bragg peaks on a linear intensity scale.

Surprisingly, the central CTR also shows local differences in the contrast, although the observed effect is much weaker than for the satellite peaks P1 and P2. This is demonstrated in Fig. 6 ▸(*a*) for the (113)_pc_ Bragg reflection, where again areas A and B can be resolved. While the contrast in P1 and P2 maps (Figs. 4 ▸ *c* and 4 ▸ *d*) is produced by the different domain alignment of the 0 and 90° variants, the contrast mechanism of the central CTR is not completely understood yet. Presumably, it arises from the different vertical lattice parameters in the 0 and 90° variants. The maximum of the CTR of the 90° domain variant appears at a slightly smaller *Q_z_* value than that of the 0° variant. This effect is observed for both the (113)_pc_ and 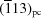 Bragg reflections and the corresponding intensities are displayed in Figs. 6 ▸(*b*) and 6 ▸(*c*). The apparent peak shift proves a slightly enlarged vertical lattice parameter by about (Δ*d*/*d*)_*z*_ = (6 ± 1) × 10^−4^ for the 90° domain variant compared with the 0° domain variant.

We can interpret the observed vertical lattice parameter difference by a simple model: for the 0° variant the corresponding in-plane lattice strains are given by ∊_*xx*_ = 0.05% and ∊_*yy*_ = −1.47% [see also Fig. 1 ▸(*b*)], whereas for the 90° variant we obtain ∊_*yy*_ = 0.07% and ∊_*xx*_ = −1.49%. However, although the net in-plane strains 〈∊〉 = ∊_*xx*_ + ∊_*yy*_ are identical for both domain variants, the elastic strain energy density *F*(∊) slightly favours the 0° variant (Fig. 1 ▸ *a*). As already stated, this energy difference can be attributed to the elastic anisotropy of the epitaxial layer, likewise leading to different vertical lattice parameters for each domain variant. Indeed, the calculations predict a slightly larger vertical lattice parameter for the 90° variant, in agreement with the experimental findings. However, the calculated numerical value of (Δ*d*/*d*)_*z*_ = 2.4 × 10^−4^ is smaller than the experimental value of 6 × 10^−4^. Nevertheless, our experimental results demonstrate that linear elasticity theory is qualitatively suitable for the correct prediction of the surface unit-cell orientation and consecutive strain field in K_*x*_Na_1−*x*_NbO_3_ epitaxial layers. In particular, our calculations

(i) indicate a preferential (001)_pc_ surface orientation of the pseudocubic unit cell;

(ii) predict that the 90° domain variant appears with lower probability than the 0° variant; and

(iii) show a larger vertical lattice parameter in the 90° variant than in the 0° variant.

The enhanced vertical strain in the 90° variant may influence the macroscopic ferroelectric properties. It is known that (epitaxial) strain has a strong impact on the Curie temperature *T*_C_. This has been theoretically demonstrated, for example, for SrTiO_3_ (Haeni *et al.*, 2004 ▸). In this material system, a compressive strain of about 6 × 10^−4^ would lead to an increase in the Curie temperature of about Δ*T*_C_ ≃ 14 K. Therefore, a difference in Curie temperature between the 0 and 90° domain variants might be expected. Future temperature-dependent investigation of ferroelectric phase transitions in this material system would thus be a very interesting topic.

## 5. Conclusions   

In summary, we have presented a scanning X-ray diffraction study of an anisotropically strained K_0.75_Na_0.25_NbO_3_ epitaxial layer using a focused monochromatic X-ray beam. With this technique, we were able to investigate the highly periodic one-dimensional ferroelectric domain pattern with a spatial resolution of about 100 nm. Besides the dominant 0° domain variant, where the domains are aligned along the 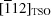 direction of the underlying (110) TbScO_3_ substrate, we were able to identify the 90° domain variant, which is aligned in the orthogonal lateral 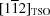 direction. Owing to the larger elastic strain energy, the 90° domain variant should appear with a rather lower probability than the dominant 0° variant, and indeed the 90° domain variant shows up as a sharp needle-shaped area in scanning X-ray micrographs. Although the net in-plane strain of the two domain types is identical, the 90° variant shows a slightly larger vertical lattice spacing. This difference can be explained by the elastic anisotropy of the K_0.75_Na_0.25_NbO_3_ epitaxial layer and can be qualitatively confirmed by calculations based on linear elasticity theory.
